# Parents’ aversion to the possibility of having a gay or lesbian child predicts gendered parenting

**DOI:** 10.1371/journal.pone.0338209

**Published:** 2025-12-04

**Authors:** Noya Kislev, Tamar Saguy

**Affiliations:** Baruch Ivcher School of Psychology, Reichman University Herzliya, Israel; Ariel University, ISRAEL

## Abstract

Gendered parenting refers to parents’ tendency to raise their sons and daughters in accordance with gender stereotypes. Previous research showed that parents’ binary conceptions of gender are associated with gendered parenting. The goal of the current work was to test whether parents’ aversion to the possibility that their child might develop a same-sex orientation – an aversion rooted in a binary view of gender – can explain gendered parenting. Negative emotions towards such a possibility were identified in previous work, however rarely studied quantitatively and have yet to be linked to gendered parenting. We conducted two studies among parents of preschool children across Israel (Study 1) and the US (Study 2). Parents were asked to choose a gift for their child, through which gendered parenting behavior was assessed. We assessed known predictors of gendered parenting that are reflective of a binary view of gender (gender essentialism and gender ideology) and added parental aversion to the possibility of having a gay or lesbian child. We further asked parents to freely explain their responses to one of the gendered parenting indicators. The results, including both qualitative and quantitative analyses, showed that parents’ aversion to the possibility of having a gay or lesbian child, exclusively predicted gendered parenting, over and above predictors identified in previous work. Results are discussed in relation to relevant constructs such as heteronormativity, homophobia, and perceived masculinity.

## Introduction

*“…How you dress and what you play with early on could start a curious response and take him down a path that would later destroy him”* (a quote by one mother, participant in Study 2).

The parent in the opening quote expresses a general aversion to the possibility that her child might develop same-sex orientation. Such aversion, which we label Parental Aversion to the Possibility of Having a Gay Child (PAGC), can have various implications for parents’ behavior. In the current work, we focus on the association between PAGC and gendered parenting, namely, parents’ tendency to promote gender typing of their young children.

Children begin to display gender-stereotypical thoughts and behaviors as early as age three [1]. This phenomenon, referred to as gender typing, reflects the process by which children acquire gendered preferences, behaviors, and roles considered appropriate for their sex [2]. Gender typing is shaped through a dynamic interaction between children’s biological predispositions, their cognitive development, and the broader social environment – including cultural norms, peer expectations, and influences from multiple socializing agents [3–5]. Among these agents, parents play a central role in shaping children’s understanding of gender [6–8]. Yet parents differ in the extent to which they encourage behaviors that align with traditional gender norms. Our research focuses on this variation, referred to as gendered parenting [9,10], and seeks to identify whether the concern that one’s child will develop same-sex orientation plays a role in predicting gendered parenting.

Gendered parenting is reflected in explicit and implicit messages and behaviors on part of parents that convey stereotypical information about how girls and boys are supposed to think, feel, and behave [10,11]. Even though gendered parenting can have significant consequences for children’s development [12–14], little work has focused on its underpinnings. The current research joins recent attempts to understand what underlies gendered parenting [9,15] by suggesting that PAGC plays a key role in driving it.

Previous work, which was mainly qualitative, identified negative emotions associated with parents’ fear that their child would incur the social sanctions associated with same-sex orientation. For example, an interview study found that parents often react to their child’s same-sex orientation with fear about their child incurring hostile and biased reactions from meaningful others, like teachers and friends, and with concerns about rejection and harm [16]. Another study among parents of adolescents who “came out” as gay or lesbian documented a parental fear of losing their child to a subculture that they could never be part of and grieving the lost opportunity to serve as role models for their children [17]. Furthermore, parents of gay or lesbian children reported struggling with their own identity as a parent of a gay child, dealing with the loss of both their children and themselves as “normal” [18]. Thus, for the most part, negative emotions associated with one’s child’s same-sex orientation have been studied as a response to a child coming out as gay or lesbian. In the current work, we propose that a related aversion – that one’s child might potentially develop a same-sex orientation – can explain why parents behave towards their children in ways that reinforce traditional gender stereotypes, referred to as gendered parenting [9].

### The binary conception of gender

Our focus in this work is on parents’ fear regarding their child’s future sexual orientation – that is, the sex of individuals their child may be romantically or sexually attracted to in the future. Even though sexual orientation is distinct from one’s gender identity (i.e., a person may identify as a man, a woman or as non-binary, regardless of whom they are attracted to ([19–24]; APA, 2015), lay conceptions of gender identity and sexual orientation are often conflated in people’s minds, reflecting a persistent reliance on a binary view of gender [22,25].

According to such binary view, humans belong to one of two dichotomous and distinct categories, girl/woman or boy/man, and belonging to one of these categories has social consequences that encompass almost every aspect of a person’s life [25, 26], including his or her sexual orientation. Indeed, from an early age, girls and boys are expected to display and adopt distinct traits, interests, and roles [27]. Girls are expected to be nurturing, sensitive, and oriented toward others [28, 29], whereas boys are expected to be strong, independent, and competitive [28,30]. These expectations are reinforced in adolescence and adulthood through traditional gender ideologies, which prescribe men to be assertive and pursue leadership roles, and women to be caring and domestically oriented [31–34]. Moreover, traditional masculinity is closely tied to heterosexuality in men, and femininity to heterosexuality in women [35–38], such that being attracted to the other sex is seen as an inherent component of being a “true” man or woman.

Because gender identity and sexual orientation are intertwined in people’s minds and correspond to a binary framework, non-traditional gender behavior might be seen as a potential sign, or even a cause, of a non-heterosexual orientation [39, 40]. Consequently, some parents may respond with heightened aversion to the possibility that their child will develop a same-sex orientation. This aversion is at the heart of the current work, which aims to show that such negative feelings, in turn, can predict parents attempts to reinforce traditional and binary conceptions of gender, i.e., enact gendered parenting practices, aiming to restore what they perceive as a normative developmental trajectory.

It is important to emphasize that we acknowledge the broad spectrum of gender identities and sexual orientations, and fully reject negative attitudes or emotions toward LGBT individuals. Our goal is not to support binary perspectives on gender, but rather to explore how these views – when adopted by parents – may influence their socialization practices in ways that sustain, and thus feed-back to reinforce, binary gender norms.

### Gendered parenting

Parents communicate expectations and norms to their children in various ways. For example, in how they dress them at an early age, through the choice of activities for them, and in the explicit statements regarding what is appropriate, or not, for girls or boys. Indeed, research on gendered parenting focuses on parental behaviors and practices that communicate, either explicitly or implicitly, gender-typed expectations. Given the dominance of play activities in the preschool age, much of the research on gendered parenting in early childhood capitalized on toys and related play [11]. For example, parents were shown to have gender-stereotypic preferences for their children’s toys [14,41]. Even prospective parents showed intentions to purchase gender-stereotypic toys for their unborn children [42]. Consistently, interaction studies reveal that parents tend to encourage (e.g., by being more engaged) play behaviors that are consistent with gender stereotypes and discourage stereotypically inconsistent behaviors [13,43]. Research further shows that young children pick up on such stereotypic preferences. For example, when asked to rate whether their parents would approve certain toys, preschoolers expected their parents to have gender-typed expectations [44].

While existing research has mostly focused on the manifestations of gendered parenting, less is known about its underlying mechanisms. Such a focus on mechanisms is important both from a theoretical and an applied point of view, since it can meaningfully contribute to the understanding of the phenomenon and of how to undermine its negative implications. Indeed, even though gender differences among young children can be partially attributed to biology and natural inclinations [3], there is also solid evidence for the impact that the immediate context can have on children’s skills and interests. For example, providing 6-year-old girls with a 20-minute programming experience reduced a gender gap favoring boys in interest in technology [45, 46]. In another study, after briefly playing with Barbie (versus Mrs. Potato Head), preschool age girls reported that boys could do significantly more male-dominated occupations than they could themselves [47].

Other work has similarly shown that children’s stereotypes about toys and occupational roles are formed early and shape their interests accordingly [48, 49], and that gendered beliefs about intellectual ability – such as the association of brilliance with men – emerge as early as age six and already influence children’s interests and aspirations [50]. These findings underscore the pivotal role of socialization processes in shaping such outcomes, and of parents as major socialization agents.

### Predictors of gendered parenting: a theoretical framework

A recent review suggested that gender-related views, including implicit and explicit gender stereotypes, gendered attributions and gender essentialism, are associated with gendered parenting [15]. For example, stronger implicit gender stereotypes among fathers predicted greater physical control behaviors toward sons [51], and mothers’ gendered attributions regarding risk behavior were linked to differential supervision of boys and girls [52]. Similarly, mothers with stronger implicit gender stereotypes emphasized traditional gender norms when interacting with their children [53], while mothers with more egalitarian gender attitudes made more counter-stereotypical remarks [54]. Consistent with these findings, traditional gender-role attitudes were found to correlate with parental discomfort toward children’s gender nonconformity [55].

Building upon these findings, recent work [9] applied a theoretical model, the Gender-Binary Cycle [25], to the context of gendered parenting. According to this model, essentialist beliefs about gender – that is, the binary view that girls/women and boys/men as fundamentally different “kinds” of humans due to inherent and unchangeable biological differences [48]), drive a non-egalitarian gender ideology, according to which men and women are viewed as fitting to different domains. Applied to the domain of parenting, this ideology implies that boys and girls should be raised in distinct ways to align with future gender roles. In turn, a non-egalitarian gender ideology gives rise to behaviors that reinforce gender stereotypes and binary conceptions of gender. Such behaviors, for example, purchasing gender stereotypic toys for one’s child, work to reinforce essentialist views of gender, which strengthen this binary cycle.

Experimental evidence supports this sequence, showing that priming individuals with gender essentialist beliefs increases gender stereotypical thinking about both the self and others [56–59]. Moreover, essentialist views of gender contribute to broader justifications of gender inequality [60, 61]. Applied to parenting, these processes suggest that parents who view gender in essentialist terms, are more likely to endorse traditional ideas about appropriate roles for boys and girls, ultimately promoting gendered socialization practices [62].

In our recent work, we further demonstrated that individuals who are motivated to sustain group-based hierarchy also have stronger essentialist views of gender, and thus are more likely to engage in gendered parenting [9]. Individuals differ in the extent to which they accept group-based hierarchy and believe that intergroup inequality is a desirable aspect of social order [63]. Previous research has shown that people high in such orientation (assessed with the Social Dominance Orientation (SDO) scale; [64]) are motivated to maintain and bolster social hierarchy [65], also in the domain of gender [66, 67], which serves to justify the division of men and women into distinct social roles of differing status and power [68–70]. In keeping with this, we found that social dominance orientation was associated with gender essentialism. Moreover, gender essentialism was associated with rigid and stereotypical perceptions about how to raise boys versus girls, which in turn was associated with gendered parenting behaviors [9].

### PAGC as a predictor of gendered parenting

Our previous work established a theoretically grounded pathway through which broader motivations to uphold social hierarchy are predictive of binary gender views and related binary gendered practices in parenting [9]. The current research explores another motivation which is also rotted in binary gender views and expressed via emotional negativity towards one’s child’s potential same-sex orientation. As explained above, binary view of gender includes the heteronormative expectation that men and women should be attracted to opposite-sex partners [71–74]. Thus, the fear that one’s child might not fulfill this expectation regarding sexual orientation, can motivate parents to enhance gendered parenting practices to “ensure” that their child is not deviating from the socially normative gender path – which is perceived to encompass both gender-congruent identity and heterosexuality.

Such motivation is likely further fueled by parents’ desire to protect their children from a difficult life trajectory. Thus, this aversion is likely intertwined with parents’ fears that their child will suffer social ostracism and rejection because of their perceived unusualness, as well as worries that the child’s life will be more difficult due to discriminatory attitudes in society towards same-sex orientation, and practical obstacles such as challenges in starting a family [16–18]. Given such concerns, parents may be motivated to encourage gender-typing and discourage deviation from stereotypic gender roles – believing these actions will help ensure their child’s future wellbeing.

In summary, the fear of a child’s social exclusion, coupled with heteronormative, binary conceptions of gender and sexuality, and a sense of responsibility for the child’s sexual identity, can all explain why PAGC might predict gendered parenting.

### Child’s sex as a moderator

Although both boys and girls are subjected to gendered parenting, evidence suggests that boys tend to encounter harsher social sanctions for deviating from gender norms [75–77]. This pattern extends to parental behaviors, with some studies indicating that parents engage in more pronounced gendered parenting practices toward boys compared to girls [55; but see 78]. For example, parents were found to give little latitude to boys’ behaviors, while encouraging both feminine behavior and masculine occupations and interests in girls [79, 80]. In another study, parents evaluated “girlish boys” more negatively than “boyish girls,” and perceived the former as more likely to have same-sex orientation than cross-gender girls [81].

Studies among LGBT youth have shown that gender-nonconforming boys encounter more negative parental reactions than gender-nonconforming girls [82], and that fathers tend to react more negatively than mothers to such gender nonconformity [83]. When parents, especially heterosexual fathers of non-conforming boys, report accepting their children’s gender counter-stereotypic behaviors, they tend to “balance” such acceptance with efforts to approximate hegemonic ideals of masculinity [84] or maintain their child’s nonconformity within gender-normative constraints [85]. Thus, among parents of boys, especially fathers, PAGC might be a stronger predictor of gendered parenting.

Finally, gender norms and attitudes toward same-sex orientation vary across cultural settings, making it valuable to examine whether the expected associations hold across distinct sociocultural contexts. Israel and the United States are both considered Western and gendered societies; however, they differ in how gender and sexuality are expressed and socially regulated. In Israel, gender distinctions are deeply embedded in the Hebrew language and reinforced by traditional and military institutions that emphasize stereotypically masculine traits [86, 87]. In contrast, public discourse in the United States tends to place greater emphasis on inclusivity and gender sensitivity, particularly in liberal circles, even though traditional expectations persist beneath the surface [88]. These similarities and differences provide a meaningful basis for comparing gendered parenting behavior and for examining whether the association between parental aversion to the possibility of having a gay or lesbian child, and gendered parenting, replicates across these two contexts.

### The current research

Study 1 was conducted among Israeli parents of preschool children, and Study 2 among American parents. Both studies were correlational and included measures of the predictors used in our previous work – social dominance orientation, gender essentialism, and gender ideology regarding child-rearing (Study 2 did not include social dominance orientation due to technical error). In both studies, we assessed PAGC using items we developed for the current research.

Across studies, the main outcome measure was the Gender Toy Choice measure, assessing parents’ actual choice of gift for their child (see details below). We also added a more explicit measure of gendered parenting by assessing parents’ reactions to their child’s hypothetical gender non-conformity (i.e., choosing a pajama that is branded counter-stereotypically). In addition, we asked parents to explain their reaction in an open-ended question. We analyzed the content of these answers to examine whether the reasons we outlined above as potentially explaining the link between PAGC and gendered parenting would be expressed by parents in their free explanations.

## Study 1

### Materials and methods

Both studies 1 and 2 were conducted in accordance with ethical guidelines for research with human participants, complying with relevant laws and institutional regulations. Ethical approval was granted by the Ethics Committee of Reichman University on December 29, 2022 (P_re1_2021156). Informed consent was obtained in writing (as part of the online questionnaire), and participants’ privacy rights were observed.

### Participants

Israeli parents were recruited using a ‘snowball’ sampling procedure initiated by twelve research assistants, undergraduate psychology students at a private university in Israel. These students shared the link to the online questionnaire with parents they knew, who, in turn, forwarded it to others within their network. Additionally, the research assistants approached parents randomly in locations frequently visited by parents of young children (e.g., playgrounds, afterschool centers). Participants were recruited between January 17, 2023, and April 13, 2023.

To ensure the independence of observations, recruiters were instructed not to recruit couples or allow the survey to be directed to a partner. Including both parents from the same family could have introduced shared variance within couples, potentially complicating the analyses without advancing the current study’s aims. Moreover, we focused exclusively on cisgender, heterosexual parents to examine gendered parenting within this population. Participants reporting non-binary gender identities or non-heterosexual orientations were excluded, as evidence suggests sexual orientation and gender identity can significantly influence parenting practices and gender socialization [89–91]. It was found that same-sex parents coparent more equally and provide less stereotypically gendered environments compared to heterosexual parents [92, 93]. Additionally, some non-binary parents practice “gender-open parenting”, refraining from assigning gender or sex to their child, though this approach is not exclusive to them [94]. Thus, while the experiences of LGBTQ+ parents merit dedicated research [95–97], they fall outside the scope of the current studies.

The initial sample included 367 participants. We excluded 17 participants who did not complete all measures, 32 participants whose child’s age fell outside the requested age range (less than 2.5 years old or older than 7 years), 3 participants who got 2 attention items wrong, 1 participant who indicated a non-binary gender identity, and 27 participants who indicated a non-heterosexual sexual orientation (some participants overlapped across these exclusions). This resulted in a final sample of 296 participants, of whom 118 were fathers (*Mean age* = 38.62, *SD* = 8.65) and 178 were mothers (*Mean age* = 36.89, *SD *= 4.87). Among the fathers, 74 (63%) responded regarding their son (*Mean age* = 4.80, *SD* = 1.32), and 44 (37%) responded regarding their daughter (*Mean age* = 4.54, *SD *= 1.09). Among the mothers, 80 (45%) responded regarding their son (*Mean age* = 4.82, *SD* = 1.25), and 98 (55%) responded regarding their daughter (*Mean age* = 4.85, *SD* = 1.20).

### Measures and procedure

Participants received a link to an online study. Upon entering the link (and after giving their consent), they were first asked to choose a gift for their child, and then they were presented with the pajama scenario item. These constituted the gendered parenting measures of the study (elaborated below). Next, participants completed scales assessing support for group-based hierarchy, gender essentialism, child-related gender ideology, PAGC, and demographic information (age, sex, gender, SES). Unless otherwise indicated, all items were rated on a 1 (not at all) to 7 (very much) scale. Full measures are presented in the online supplementary materials (SM).

### The gendered toy choice

We developed a behavioral measure of gendered parenting to enable an unobtrusive assessment and avoid demand characteristics. The measure was validated in two studies among independent samples of parents of preschool children [98] and has been used in three additional studies [9].

After providing their consent to participate, parents were presented with a set of toys and were told that, in appreciation of their participation, they would enter a raffle to win a toy prize for their child. They were then asked to indicate their child’s sex, age, and to choose a toy for their child from 20 toy options offered to them. The set of toys varied in gender typicality, based on a pilot study in which toys were rated on a nine-point scale ranging from 1 (extremely feminine) to 9 (extremely masculine), with 5 indicating “gender neutrality” (see SM, Table 1, for the full list with scores). The selection of toys by participants was conducted under the premise of a real choice scenario. Parents were informed that the raffle winners would receive the actual toy they selected, enhancing the authenticity of their decisions. However, for logistical reasons, the winners ultimately received vouchers for online purchase of toys of equivalent value. Importantly, this modification was not disclosed to the parents prior to or during the selection process, thereby preserving the integrity of their choices.

**Table 1:** Means, SDs, and correlations among the main variables (Study 1).

		M	SD	1	2	3	4	5	6	7	8
1	**Child’s Sex**	–	–								
2	**Parent’s Gender**	–	–	.17**							
3	**SDO**	2.32	.87	−.04	−.16**						
4	**Gender Essentialism**	3.74	1.05	−.12*	−.11	.35**					
5	**Gender Ideology**	2.43	.88	−.04	−.38**	.47**	.41**				
6	**PAGC**	3.54	1.48	−.07	−.16**	.29**	.32**	.46**			
7	**Wanted Gifts**	6.34	1.58	−.08	−.16**	.08	.14*	.08	.16**		
8	**Unwanted Gifts**	3.65	2.48	.46**	.16**	−.01	−.16**	−.10	−.16**	−.37**	
9	**Pajama Scenario**	2.84	1.59	−.31**	−.13*	.20**	.30**	.30**	.45**	.16**	−.28**

*Correlation is significant at the 0.05 level (2-tailed)

**Correlation is significant at the 0.01 level (2-tailed)

To capture both the encouragement of gender-stereotypic expressions and the discouragement of counter-stereotypic expressions with this behavioral measure, participants were asked to list two options for the most preferred toys and two options for the least preferred toys. Thus, gendered parenting was assessed using two index scores: (1) the Wanted Gifts Index, calculated as the mean gender typicality rating of the two preferred toys. The gender typicality scale was reversed for girls. Thus, for both girls and boys, a higher score indicated more gender-typed toy choice; (2) the Unwanted Gifts Index, calculated as the mean gender typicality rating of the two least-preferred toys. The gender typicality scale was also reversed for girls. Thus, for both girls and boys, a lower score corresponded to less gender typicality of the toy. Since this was the least preferred gift, a lower score indicated higher gender-typed behavior by the parent.

### Pajama scenario

Based on Spivey et al. [55], we devised a scenario describing a non-conforming gendered behavior in which a child chooses a counter-stereotypical pajama design as a birthday present. Parents were asked to report the extent to which they would encourage or discourage their child’s counter-stereotypical choice, providing a direct measure of gendered parenting behavior, while a higher score represented a more gendered behavior by the parent. Additionally, participants were invited to explain their responses and freely express their thoughts on this issue through an open-ended question. This qualitative information was used in subsequent analyses.

### Endorsement of social hierarchy

Support for group-based hierarchy was assessed using the adapted Social Dominance Orientation Scale (SDO; [64]), which included 8 items (e.g., “It’s probably a good thing that certain groups are at the top and other groups are at the bottom”; “Some groups of people are just inferior to others”; *Cronbach’s α = .73*). A higher score represented a stronger support of social hierarchy by the parent.

### Gender essentialism

Gender essentialism was assessed using a shortened version of the Gender Essentialism Scale (GES; [99]), consisting of 7 items (e.g., “Differences between women’s and men’s personalities are in their DNA”; “Male and female brains probably work in very different ways”; *Cronbach’s α* = .78). A higher score represented more endorsement of essentialist views of gender by the parent.

### Child rearing gender ideology

Gender ideology with respect to child rearing was assessed using 8 items adapted from the Child Rearing Sex-Role Attitude Scale (CRSRAS; [100]). Parents were asked to indicate the extent to which they agree with statements regarding raising children, such as: “Boys who exhibit sissy behaviors will never be well adjusted”; “Quiet girls will have a happier life than assertive girls”; *Cronbach’s α* = .75. A higher score represented a stronger support in non-egalitarian gender ideology regarding child rearing. This scale has been used in previous research to assess gender-typed attitudes towards rearing girls and boys [13,44,101,102].

### Parental aversion to the possibility of having a gay or lesbian child (PAGC)

To assess this construct, we developed 5 items in which parents were asked to rate the extent to which they might experience negative emotions to the possibility of having a gay or lesbian child. Specifically, participants responded to the following items (on 1–7 scale): “People have a variety of feelings and thoughts about sexual orientation. If your child had homosexual tendencies, to what extent would you be (a) worried or not worried? (b) disturbed or not disturbed? (c) upset or not upset? (d) happy or not happy? (e) resentful or not resentful?” Responses were averaged to create a composite score, with the “happy” item reverse-coded (*Cronbach’s α* = .88). A higher score represented a stronger aversion to the possibility that one’s child will have same-sex orientation.

### Results

#### Analyses strategy.

Our goal was to examine whether PAGC significantly contributed to the explained variance in gendered parenting indices, beyond predictors identified in previous work on gendered parenting, referred to as known predictors hereafter (social dominance orientation, gender essentialism, and child-rearing gender ideology). We used hierarchical regression analysis, with the known predictors: social dominance orientation (SDO), gender essentialism and child-rearing gender ideology, and demographics (parent’s gender, child’s sex, parent’s age and subjective socio-economic status (SES)) entered in Block 1. Block 2 included the interaction between child’s sex and parent’s gender. PAGC was entered as an exclusive predictor in Block 3. In Block 4, we added the interaction between child’s sex and PAGC.

Table 1 presents the means, SDs, and correlations among all main variables.

Next, we conducted the hierarchical regression as specified above, testing the model three times, with each of the gendered parenting indices – wanted gifts, unwanted gifts, and the pajama item. Table 2 presents the blocks in the hierarchical regression model predicting gendered parenting indices among Israeli Parents.

**Table 2:** Blocks in the hierarchical regression model predicting wanted gifts, unwanted gifts, and pajama indices among Israeli parents (Study 1).

	Wanted Gifts			Unwanted Gifts			Pajama		
Predictor	*B (SE)*	*t*	*p-value*	*B (SE)*	*t*	*p-value*	*B (SE)*	*t*	*p-value*
**Block 1**									
SDO (1–7)	.01 *(.12)*	.18	.86 (n.s.)	.09 *(.17)*	1.50	.13 (n.s.)	.02 *(.11)*	.38	.70 (n.s.)
Gender Essentialism (1–7)	.13 *(.10)*	1.98	<.05	−.10 *(.14)*	−1.75	.08 (n.s.)	.17 *(.09)*	2.85	<.01
Child rearing Gender Ideology (1-7)	−.05 *(.13)*	−.64	.52 (n.s.)	−.06 *(.19)*	−.88	.38 (n.s)	.22 *(.12)*	3.29	<.01
Parent’s Gender (1 = men; 2 = women)	−.16 *(.20)*	−2.46	<.05	.08 *(.29)*	1.34	.18 (n.s.)	.01 *(.19)*	.15	.89 (n.s.)
Child’s Sex (1 = boy; 2 = girl)	−.05 *(.19)*	−.87	.39 (n.s.)	.43 *(.26)*	8.10	<.001	−.27 *(.17)*	−5.07	<. 001
Parent’s Age	−.10 *(.01)*	−1.66	.10 (n.s.)	.08 *(.02)*	1.48	.14 (n.s.)	−.07 *(.01)*	−1.30	.19 (n.s.)
SES	−.07 *(.13)*	−1.18	.24 (n.s.)	−.07 *(.18)*	−1.27	.21 (n.s.)	.08 *(.12)*	1.55	.12 (n.s.)
**Block 2**									
Child’s Sex × Parent’s Gender	.32 *(.38)*	1.12	.27 (n.s.)	.01 *(.54)*	.04	.97 (n.s.)	.41 *(.35)*	1.58	.11 (n.s.)
**Block 3**									
PAGC (1–7)	.15 *(.07)*	2.21	<.05	−.10 *(.10)*	−1.68	.09 (n.s.)	.34 *(.06)*	6.04	<.001
**Block 4**									
Child’s Sex × PAGC	−.08 *(.12)*	−.34	.73 (n.s.)	.07 *(.18)*	.36	.72 (n.s.)	−.54 *(.11)*	−2.88	<.01

### Wanted gifts

For the wanted gifts index, Block 1 was significant (*R²* = .06, *p *= .02), with gender essentialism and parent’s gender as significant predictors (see Table 2), such that fathers (versus mothers) and parents reporting higher levels of gender essentialism chose more gendered gifts. The interaction between child’s sex and parent’s gender was not significant (Block 2). PAGC was found to be a significant predictor, as well as the change from Block 2 to Block 3 (*ΔR²* = .02, *p* = .03), indicating that the more parents scored higher on the PAGC measure, the more they chose gendered gifts. The interaction of child’s sex × PAGC (Block 4) did not add significant explained variance. The entire model explained 7.8% of the variance in the wanted gifts index.

### Unwanted gifts

For the unwanted gifts index, Block 1 was significant (*R²* = .24, *p *< .001), with child’s sex as significant predictor (see Table 2), meaning parents to boys (versus parents to girls) were more likely to avoid gender counter-stereotypical gifts. The interaction between child’s sex and parent’s gender (Block 2) was not significant nor the change from Block 1 to Block 2. PAGC (Block 3) also did not add significant explained variance in the unwanted gifts, nor did the interaction child’s sex × PAGC (see Table 2).

### Pajama item

For the pajama item, Block 1 was significant (*R²* = .21, *p* < .001), with gender essentialism, child-rearing gender ideology and child’s sex as significant predictors (see Table 2), such that parents to boys (versus parents to girls) and parents reporting higher levels of gender essentialism and less egalitarian gender ideology were more likely to resent a counter-stereotypical choice of pajama by their child. The interaction between child’s sex and parent’s gender was not significant (Block 2). PAGC (Block 3) was a significant predictor, as well as the change from Block 2 to Block 3 (*ΔR*^*2*^ = .09, *p* < .001), indicating that the more parents scored higher on the PAGC measure, the more they resented their child’s choice of counter-stereotypic pajama. The interaction child’s sex × PAGC (Block 4) added significant explained variance in the pajama item (*ΔR*^*2*^ = .02, *p* < .01). The entire model explained a total of 32.9% of the variance in this index.

To follow up on the interaction, we examined the effect of PAGC on the pajama scenario item among parents of sons and parents of daughters separately, controlling for all the variables entered in Step 1 (parent’s demographics: gender, age, SES, and SDO, gender essentialism and gender ideology). PAGC was a significant predictor of the pajama item among parents of sons, *β *= .43, *p* < .001, as well as among parents of daughters, *β *= .27, *p* < .01, although the effect was stronger for boys. Thus, for parents to sons (versus daughters), the aversion to the possibility that their child will develop a same-sex orientation was a stronger predictor of parental resentment of counter-stereotypic behavior.

### Analyses of open-ended responses

We examined parents’ open-ended responses to the question asking them to explain their reaction to the pajama scenario item. We were particularly interested in the reasons expressed by parents who indicated strong opposition to the counter-stereotypical choice of pajama, expecting that such reasons might reflect PAGC (Parental Aversion to the possibility of having a Gay or lesbian Child) and related concerns. Given that parents’ responses to the pajama scenario were strongly associated with their PAGC (10% of shared variance), these explanations can be used to better understand the association between PAGC and gendered parenting.

### Parents of boys

A total of 66 parents of boys – 25 fathers and 41 mothers – chose to elaborate on their choice of response to the pajama scenario item. Table 3 presents examples/themes of these explanations.

**Table 3:** Examples/ themes of parents’ explanations of their response to the pajama item (Study 1).

	Parents of Boys		Parents of Girls	
Pajama Scenario Score	% of parents	Explanation Examples/themes	% of parents	Explanation Examples/themes
**5-7** **(most gendered response)**	44%	(1) “It’s a girl’s pajama”; “It’s inappropriate for boys”; “the colors and design are feminine, more suitable for girls”; “I want to stress the differences between boys and girls”. (2) “As a parent, it’s my responsibility to guide my son about the social norms”; “I should teach my son about acceptable gender norms”, “I don’t want my son to be misdirected towards feminine gender norms”. (3) “I would much prefer if my son conforms with the mainstream, at least outside the house”; “I’m afraid that my child’s counter-stereotypical choice would cause other children to mock him; “I am trying to prevent my son from suffering insults and unpleasant emotions, due to others reactions to gender nonconformity”.	17%	(1) “It’s a boy’s pajama”; “the unicorn pajama is more suitable for girls”; “I don’t want her to be the only girl wearing this” (2) “I’m afraid that my daughter will be mocked by the other children for choosing a boy’s pajama”; “she will be bothered if she gets comments on her extraordinary choice”; “young children can be very judgmental and I would not want her to be rejected because of that”
**4 (mean)**	10.5%	“I don’t care, my son can choose whatever he feels like”; “I approve of any choice that would make my son happy”; “at this age, children should be given free choice in such matters”	8.5%	“She can choose whatever she wants”; “I really don’t care what she chooses. It doesn’t define her”; “There is no need to make a big deal out of it”
**1-3 (least gendered response)**	45.5%	“I accept and will support every choice of my son”; “I’m happy with whatever makes him happy”; “I encourage my son’s individual free choice”; “I’m pleased that my son does not conform to gender norms”	74.5%	(1) “I’m o.k. with whatever my daughter desires”; “I don’t mind, she has to wear it” (2) “As long as my daughter is happy and feels comfortable, any choice of her is good by me”; “I support her to choose whatever she likes and do whatever she wants, regardless the social norms”; “different colors and designs can be for everybody”. (3) “I let my daughter choose for herself, and she has super-heroes pajamas at home”; “I encourage gender non-conformity and will be happy if she expresses independent choice”.

Parents who scored high on the pajama item emphasized binary perceptions of gender in their explanations and expressed a desire to instill these perceptions in their children, as these views are considered prevalent and most accepted in society (“I want to stress the differences between boys and girls”). These reactions align with heteronormative views of gender. Additionally, these parents expressed explicit fears that children who behave in a gender-nonconforming manner will suffer insults and be ridiculed (“I am trying to prevent my son from suffering insults and unpleasant emotions”). These views correspond to the reasons presented in the introduction for why PAGC is a predictor of gendered parenting.

In contrast, parents who obtained a neutral score on the pajama item emphasized giving the child the freedom to choose, considering the child’s young age, and overall did not attribute much significance to such a choice of clothing. Finally, among parents who obtained the lowest scores, the explanations not only included encouragement of free choice by the child but also expressed the promotion of individuality by the parent, with a few cases even explicitly supporting non-conformity. These reasons reflect the opposite of the views held by parents who scored high on this item.

### Parents of girls

A total of 58 parents of girls – 14 fathers and 44 mothers – chose to elaborate on their choice of response to the pajama scenario item. Table 3 presents examples/themes of these explanations. In general, this group of parents scored lower on this item compared to parents of boys, indicating less discomfort with their daughter wearing the counter-stereotypical pajama. The themes of the more gendered responses were similar to those reported by parents of boys, showing that heteronormative views of gender (“the unicorn pajama is more suitable for girls”) and the motivation to protect one’s child (“I’m afraid that my daughter will be mocked by the other children for choosing a boy’s pajama”) were also prominent themes among parents of girls. However, most parents of girls scored low on the item, indicating acceptance of their daughter’s gender counter-stereotypical choice. Thus, although the various themes that arose among parents of girls were substantially similar to those that arose among parents of boys (who obtained similar scores on the pajama item, respectively), it was clear that parents of girls’ responses reflected much less gendered parenting compared to parents of boys. This finding aligns with the concept of precarious manhood, which suggests that masculinity is perceived as fragile and must be actively acquired by boys (and instilled by fathers), and consistently maintained through actions that demonstrate it [103].

## Study 2

### Materials and methods

#### Participants.

Participants were recruited via an online platform (Prolific). The sample consisted of American parents who were native English speakers and had at least one child of preschool age (2.5–7 years). Participants were recruited between May 18, 2023, and June 1, 2023. In exchange for their participation, participants were compensated as customary and entered a raffle for a toy prize for their child (worth $30 USD). Their choice of toys (two most wanted and two most unwanted out of 20 offered) at the beginning of the survey served as a behavioral measure of gendered parenting. In practice, five participants (randomly chosen) received an Amazon gift card worth $30.

The sample included 453 participants, of whom 8 were excluded for missing values (particularly, they did not report their sex). Two participants were excluded for failing two attention check items, 28 additional participants for indicating a child’s age that was not within the requested range, and 14 more participants were excluded for not reporting their child’s age. A further 69 participants, who reported a non-heterosexual orientation, were also excluded (some overlapped with other excluded categories).

This resulted in a final sample of 354 parents, comprising 182 fathers (*Mean age* = 37.74, *SD* = 6.45) and 172 mothers (*Mean age* = 36.63, *SD* = 6.65). Of the fathers, 99 chose a gift for their son (*Mean age* = 4.52, *SD* = .86), and 83 for their daughter (*Mean age* = 4.42, *SD* = .87). Of the mothers, 84 chose a gift for their son (*Mean age* = 4.39, *SD* = .84) and 88 for their daughter (*Mean age* = 4.46, *SD* = .90).

### Measures and procedure

The same measures and procedures were used as in Study 1, with the following differences: (1) In Study 2, we used an additional measure of gendered parenting, where parents were asked to choose their most preferred camp activities for their child. (2) In Study 2, we used an adapted version of the Gender Toy Choice measure to suit American parents (see below). (3) In Study 2, we did not include the measure of social dominance orientation, due to a technical error in the survey programming stage, which resulted in the inadvertent omission of these items.

### The gendered activity choice

Parents were presented with a list of camp activities for children that varied in gender typicality. They were told that an organization that operates courses and summer camps for children was conducting a survey among parents, to assess their preferences for activities they would choose for their child, if the child were to enroll in the camp. Parents were asked to choose three activities in which they would like their child to participate out of 20 presented to them, assuming their child were to enroll in such a summer camp.

The activities were rated in a pretest held among a different sample of American adults to assign a gender-typicality score for each, ranging from 1 (extremely feminine) to 9 (extremely masculine). The final list of activities included four that were rated as highly masculine (e.g., football), four rated as somewhat masculine (e.g., Lego), four rated as gender-neutral (e.g., capoeira), four rated as somewhat feminine (e.g., cooking), and four rated as highly feminine (e.g., ballet dancing) (see SM). The mean of gender-typicality rating was computed for the three preferred activities chosen by the parent. As in the Gender Toy Choice measure, the gender-typicality scale was reversed for girls. Thus, for both girls and boys, a higher score indicates a preference for more gender-typed activities.

### The gendered toy choice, english version

As specified above, the Gendered Toy Choice measure (the wanted gifts and unwanted gifts indices) was developed and first validated in Hebrew among samples of Israeli parents. In Study 2, we used an adapted, validated English version of this measure [98] (see SM).

### Results

Table 4 presents means, standard deviations, and correlations among all main variables.

**Table 4:** Means, SDs, and correlations among the main variables (Study 2).

		M	SD	1	2	3	4	5	6	7	8
1	**Child’s Sex**	–	–								
2	**Parent’s Gender**	–	–	.06							
3	**Gender Essentialism**	4.17	1.14	−.07	−.05						
4	**Gender Ideology**	2.54	1.23	−.15**	−.23**	.48**					
5	**PAGC**	3.67	1.89	−.17**	−.12*	.51**	.69**				
6	**Wanted Gifts**	6.62	1.45	−.07	−.03	.10	.00	.11*			
7	**Unwanted Gifts**	3.20	2.09	.43**	.08	−.12*	−.04	−.14*	−.38**		
8	**Pajama Scenario**	3.21	1.87	−.39**	−.03	.33**	.38**	.58**	.16**	−.33**	
9	**Activity Choice**	5.51	.80	.19**	−.02	.25**	.21**	.27**	.26**	−.31**	.25**

*Correlation is significant at the 0.05 level (2-tailed)

** Correlation is significant at the 0.01 level (2-tailed)

Next, as in Study 1, we examined whether PAGC predicts gendered parenting above and beyond demographic variables and the other predictors included in the study, which were identified in our previous work. Using hierarchical regression analysis, we tested the model four times, with each of the gendered parenting indices: wanted gifts, unwanted gifts, the pajama item, and activity choice. Gender essentialism, child-rearing gender ideology, parent’s age, SES, child’s sex, parent’s gender were defined as predictors in Block 1, and the interaction between child’s sex and parent’s gender was defined as a predictor in Block 2. In Block 3, we added PAGC as a predictor, and finally, the interaction variable of child’s sex × PAGC (Block 4). Table 5 presents the blocks in the hierarchical regression model predicting wanted gifts and unwanted gifts indices among American parents.

**Table 5:** Blocks in the hierarchical regression model predicting wanted gifts and unwanted gifts indices among American parents (Study 2).

	Wanted Gifts			Unwanted Gifts		
Predictor	*B (SE)*	*t*	*p-value*	*B (SE)*	*t*	*p-value*
**Block 1**						
Gender Essentialism (1–7)	.13 *(.08)*	2.09	<.05	−.14 *(.10)*	−2.47	<.05
Child rearing Gender Ideology (1–7)	−.08 *(.07)*	−1.18	.24 (n.s.)	.11 *(.10)*	1.94	.053
Parent’s Gender (1 = men; 2 = women)	−.04 *(.16)*	−.76	.45 (n.s.)	.07 *(.21)*	.37	.17 (n.s)
Child’s Sex (1 = boy; 2 = girl)	.08 *(.16)*	−1.48	.14 (n.s.)	.43 *(.21)*	8.77	<.001
Parent’s Age	.02 *(.01)*	.42	.68 (n.s.)	−.01 *(.02)*	−.18	.86 (n.s.)
SES	−.04 *(.09)*	−.81	.42 (n.s.)	−.03 *(.12)*	−.53	.59 (n.s.)
**Block 2**						
Child’s Sex × Parent’s Gender	−.07 *(.31)*	−.41	.65 (n.s.)	−.07 *(.41)*	−.31	.76 (n.s.)
**Block 3**						
PAGC (1–7)	.17 *(.06)*	2.20	<. 05	−.13 *(.08)*	−1.91	.057 (n.s.)
**Block 4**						
Child’s Sex × PAGC	−.10 (.08)	−.51	.61 (n.s.)	.20 *(.11)*	1.17	.24 (n.s.)

### Wanted gifts

Block 1 was not significant (*R²* = .02, n.s.), as gender essentialism was the only significant predictor in this step, such that parents reporting higher levels of gender essentialism chose more gendered gifts. The interaction between child’s sex and parent’s gender was also non-significant (Block 2). In Block 3, PAGC was found as a significant predictor, as well as the change from Block 1 to Block 2 (*∆R*^*2*^* *= .01, *p* < .05), indicating that parents who scored higher on the PAGC measure were more likely to choose gendered gifts. Block 4 did not add significant explained variance, as the interaction between child’s sex × PAGC was not significant (see Table 4). The entire model explained 3.6% of the variance in this index. These results replicated Study 1, with the exception that parent’s gender was a significant predictor in Block 1 only among the Israeli sample (Study 1), but not among American parents.

### Unwanted gifts

Block 1 was significant (*R²* = .02, *p < .001*), with gender essentialism and child’s sex as significant predictors in this step, such that parents reporting higher levels of gender essentialism and parents to boys (versus parents to girls), were more likely to avoid gender counter-stereotypical gifts. Gender Ideology was not significance (*β* = .11, *p* = .053). The interaction between child’s sex and parent’s gender was also non-significant (Block 2). PAGC (Block 3) was not significant, as well as the change from Block 2 to Block 3 (*∆R² *= .01, *p* = .057). Block 4 did not add a significant explained variance (see Table 5). These results were similar to those obtained in Study 1, with the exception that gender essentialism was a significant predictor of unwanted gifts in Block 1 only among the American sample (Study 2), but not among Israeli parents.

Table 6 presents the blocks in the hierarchical regression model predicting pajama and activity choice indices among American parents.

**Table 6:** Blocks in the hierarchical regression model predicting pajama and activity choice Indices among American parents (Study 2).

	Pajama			Activity Choice		
Predictor	*B (SE)*	*t*	*p-value*	*B (SE)*	*t*	*p-value*
**Block 1**						
Gender Essentialism (1–7)	.19 *(.08)*	3.62	<. 001	.19 *(.04)*	3.17	<.01
Child rearing Gender Ideology (1–7)	.25 *(.08)*	4.78	<. 001	.09 *(.04)*	1.56	.12 (n.s.)
Parent’s Gender (1 = men; 2 = women)	.04 *(.17)*	.76	.45 (n.s.)	.02 *(.09)*	.35	.73 (n.s.)
Child’s Sex (1 = boy; 2 = girl)	−35 *(.17)*	−7.73	<.001	−.16 *(.08)*	−3.16	<.01
Parent’s Age	−.08 *(.01)*	−1.67	.10 (n.s.)	−.05 *(.01)*	−.94	.35 (n.s.)
SES	−.10 *(.01)*	−2.27	<.05	−.01 *(.05)*	−.12	.91 (n.s.)
**Block 2**						
Child’s Sex × Parent’s Gender	−.20 *(.34)*	−1.03	.31 (n.s.)	.52 *(.16)*	2.29	<.05
**Block 3**						
PAGC (1–7)	.52 *(.06)*	8.93	<.001	.19 *(.03)*	2.58	<.05
**Block 4**						
Child’s Sex × PAGC	−.29 *(.08)*	−2.04	<.05	−.29 *(.04)*	−1.63	.10 (n.s.)

### Pajama scenario item

Block 1 was significant (*R²* = .31, *p* < .001), with gender essentialism, child-rearing gender ideology, child’s sex and subjective socio-economic status (SES) as significant predictors (see Table 6), such that parents of boys and parents reporting higher levels of gender essentialism and less egalitarian gender ideology, as well as parents reporting lower socio-economic status, were more likely to resent a counter-stereotypical choice of pajamas by their child. The interaction between child’s sex and parent’s gender was also non-significant (Block 2). PAGC (Block 3) was a significant predictor, as well as the change from Block 2 to Block 3 (*∆R*^*2*^* *= .13, *p* < .001), indicating that parents who scored higher on the PAGC measure were more likely to resent their child’s choice of counter-stereotypic pajama. The interaction between child’s sex × PAGC (Block 4) added a small, yet significant explained variance in the pajama item (*∆R*^*2*^* *= .01, *p* < .05). The entire model explained a total of 44.7% of the variance in this index (see Table 6). These results resembled those of Study 1, where gender essentialism, gender ideology, child’s sex, PAGC and the interaction child’s sex X PAGC, were significant predictors of the variance in the pajama item scenario, across both Israeli and American samples.

To follow up on the interaction, we examined the effect of PAGC on the pajama item among parents of sons and parents of daughters separately, controlling for all the variables entered in Step 1. PAGC was a significant predictor of the pajama item among both parents of sons, *β* = .62, *p* < .001, and parents of daughters, *β* = .44, *p* < .001, with the effect for sons being stronger – replicating the pattern found in Study 1.

### Activity choice

Block 1 was significant (*R²* = .10, *p* < .001), with gender essentialism and child’s sex as significant predictors (see Table 6), such that parents to boys (versus parents to girls) as well as parents reporting higher levels of gender essentialism, were more likely to choose gender-stereotypic activities for their children. The interaction between child’s sex and parent’s gender (Block 2) was significant, as well as the change from Block 1 to Block 2 (*∆R*^*2*^* *= .01, *p* < .05). To follow up on the interaction child’s sex X parent’s gender in Block 2, we examined the effect of child’s sex on activity choice among mothers and fathers separately, controlling for all the variables entered in Step 1. Child’s sex was a significant predictor of the activities that were chosen by the parents, only among fathers, *β* = −.26, *p* < .001, but not among mothers, *β* = −.06, *p* = .47 (n.s.), meaning that it was the fathers that most differentiated their activities’ choice based on child’s sex. PAGC (Block 3) was a significant predictor, indicating that parents who scored higher on the PAGC measure were more likely to choose gendered activities for their child. Block 4 (the interaction between child’s sex and PAGC) did not add significant explained variance in the activity choice measure (see Table 6). The entire model explained 13.6% of the variance in the activities choice.

### Analyses of open-ended responses

#### Parents of boys.

A total of 117 parents of boys – 60 fathers and 57 mothers – chose to elaborate and explain their choice of answer in the pajama scenario item. Table 7 presents examples/themes of parents’ explanations. As in Study 1, American parents who balked at their child’s choice and chose the highly gendered responses (scored 5–7 on this item) explained their reasoning similarly to Israeli parents (Study 1). They emphasized the binary division between what is “appropriate for boys” and what is “appropriate for girls”, highlighting the importance of adhering to and teaching acceptable gender norms to their children. Some parents further elaborated, expressing a desire to protect their children from negative social experiences, which aligns with the reasons specified in the introduction regarding the association between PAGC and gendered parenting.

**Table 7:** Examples/themes of parents’ explanations of their response in the pajama Item (Study 2).

	Parents of Boys		Parents of Girls	
Pajama Scenario Score	% of parents	Explanation Examples/themes	% of parents	Explanation Examples/themes
**5-7** **(most gendered response)**	46%	(1) “I would not allow my son to wear such an overtly feminine outfit”; “This would not be an appropriate choice. He’s a boy”. (2) “I know that trans rights are a big thing right now and, while I firmly believe that every ADULT has the right to dress and act as they choose, I also know that that’s not the life I want for my child. I don’t want him growing up think that it is acceptable to dress in women’s clothing because it’s not. I don’t care what others do. I will never treat anyone any differently for the choices they make but I don’t want that kind of life for my son. I don’t want the stigma that comes with it and I don’t want the difficulties that come with it. If he makes different choices when he’s grown, then those are his choices. At least I will know that I guided him in the right direction”	10%	(1) “There are certain clothes that boys should wear and there are certain close that girls should wear”; “I’m raising a daughter as a girl. Not in a way to imitate boys”; (2) “I would try to have her choose the outfit socially acceptable for her gender”; “I just think it would be better if acted and behaved in a feminine way”.
**4 (mean)**	7%	“He can wear whatever makes him happy”; “He’s in kindergarten, I don’t think it really matters”; “I would probably roll with it at his age”; “Still at a young age so I would not place much concern here”.	13%	“I don’t think it’s a big deal”; “I am indifferent about the selection. At a young age, kids gravitate to what they like regardless of societal gender norms. I will not make a fuss about it and be ok with their decision”; “I don’t mind either way. As long as she’s happy” “I’d suggest the girly pajamas but if she ultimately wanted the boyish one, I would let her have them”.
**1-3 (least gendered response)**	47%	(1) “I think kids having the ability to express their likes and interests is important. Just because I may have not chosen the “more feminine” choice for my son doesn’t mean I have the right to take that choice away from him. He’s his own person and should be able to choose his own likes and express himself as he pleases”. (2) “I am fine with him wearing whatever he wants, I’m afraid other parents don’t teach their kids to be kind and open minded. He is a sweet soul, and I wouldn’t want him made fun of”“ (3) “Pink and unicorns, while leaning towards feminine, aren’t so girly to be unacceptable for him to wear. I would absolutely allow it and be excited for him if he picked it on his own. I might tell him that pink is associated with girl clothes and see if he wants to change his mind, but I wouldn’t force it”. “I think it is important to let a child fully express their likes and interests and not try to shape them into your own expectations. I would explain that some other children may ask things like “why are you wearing girl clothes” but that is not because he is doing something wrong, but because that is what their family taught them, and it is ok for boys and girls to like the same things”.	77%	(1) “I feel like children are little people that have their own thoughts and ideas about what they like. It’s my job to raise her into a thoughtful, empathic, and kind adult. It’s not my job to tell her who she is or what she likes. She is free to figure that out on her own. I’ll love her regardless”. (2) “I don’t think that the preferences of my preschooler need to be changed at all and if she likes boyish things then she likes them, I don’t understand people who force children to wear something that they don’t want to wear”. “As long as my daughter is happy and feels comfortable, any choice of her is good by me”; “I support her to choose whatever she likes and do whatever she wants, regardless the social norms”; “different colors and designs can be for everybody”. (3) “I let my daughter choose for herself, and she has super-heroes’ pajamas at home”; “I encourage gender non-conformity and will be happy if she expresses independent choice”.

Moreover, it appears that in Study 2, these parents expressed even stronger reluctance regarding the non-conforming choice, using harsher and more explicit language to convey their disapproval (e.g., “I would not allow my son to wear such an overtly feminine outfit”; “I would not want him to wear girls’ clothing. It is not right”). The explanations provided by parents encapsulate three themes that seem to be at the core of the association between PAGC and gendered parenting: heteronormativity, protecting one’s child from a hostile environment, and feeling responsible for their child’s sexuality.

Parents who chose the neutral score of 4 were characterized by similar main themes as the Israeli parents, reflecting indifference to their child’s choice. Finally, parents who scored 1–3, who supported their son’s counter-stereotypical choice of unicorn pajamas, mostly expressed similar reasoning to the Israeli parents (Study 1), emphasizing free choice, support of individualism, and even non-conformity. However, some parents, while approving their son’s choice, also expressed concerns regarding social responses and mockery. Other parents, although they reported an open, accepting attitude toward the “feminine” choice, still felt the need to educate their sons about traditional gender norms. Furthermore, there were parents who stated they would warn their sons of possible social backlash they might experience because of this choice.

Thus, among parents of boys, even in the least gendered category of scores, parents’ explanations reveal an ambivalent attitude towards a “feminine” act or expression by their son. Stated differently, even when allowing the counter-stereotypical act, these parents were still concerned about social backlash or felt the need to maintain their child’s nonconformity within gender-normative constraints [84,85]-.

#### Parents of girls.

A total of 128 parents of girls – 62 fathers and 66 mothers – chose to elaborate and explain their choice of answer in the pajama scenario item. Table 7 presents examples/themes of parents’ explanations. Overall, parents of girls (*M* = 2.46, *SD* = 1.38) scored lower than parents of boys (*M* = 3.92, *SD* = 1.98) on this item, *t*(352) = 8.00, *p* < .001. Parents who chose the highly gendered responses explained their reasoning similarly, emphasizing the need to preserve distinct spheres for boys versus girls and to conform to what they believe is socially accepted. Parents who chose the neutral score of 4 were characterized by similar main themes compared to parents of boys and Israeli parents in general – emphasizing their children’s happiness with whatever they choose and the belief that at this young age, it is not a problem. However, a few parents stated that they would have preferred the “girlish” pajama for their daughter, though they would eventually let her choose. Finally, parents who scored low on this item, who supported their daughter’s counter-stereotypical choice of cars pajama, based their reasoning on similar principles to parents of boys and Israeli parents (Study 1), expressing mostly liberal values such as freedom and acceptance of individuality.

Thus, comparing parents of girls’ responses to those of parents of boys, pointed to a clear trend, of which parents of boys enacted in more gendered parenting compared to parents of girls – in the sense that parents of boys expressed greater disapproval of their son’s choice of a counter-stereotypical pajama, and used harsher, and more explicit wording while explaining their response. Among the main reasons for their disapproval, parents mentioned binary and conservative perceptions of gender and gender roles, aversion towards deviation from socially accepted gender norms, and fear of ridicule and social ostracism that might be directed at their child – all corresponding with the reasons presented in the introduction.

## General discussion

In this work, we aimed to explain parents’ tendency to promote the gender-typing of their young children. Across two studies conducted in Israel and the United States, the data supported the unique contribution of the PAGC construct to the explained variability in gendered parenting, above and beyond some demographic variables (child’s sex, parent’s gender, parent’s age, SES) and several other variables (social dominance orientation, gender essentialism and gender ideology regarding child rearing) identified in our previous work [9], and by others (e.g., [15,62,99]). Results showed that among both Israeli and American parents, PAGC (and in some cases, also its interaction with child’s sex) significantly predicted gendered parenting practices, as measured both implicitly (with the Gender Toy Choice measure and to a lesser extent, with the activity measure) and explicitly with the pajama item. Moreover, in their verbal explanations for their ratings in the pajama item, parents who most disapproved of their child’s non-conforming choice, expressed the strongest resistance to deviation from the gender binary; the necessity to educate their children about socially acceptable gender norms and roles; and the desire to protect them from social rejection. These themes were the very reasons we hypothesized that PAGC would predict gendered parenting.

However, it should be noted that parental aversion to their child’s potential same-sex orientation constitutes only one among multiple factors that may shape gendered parenting practices. Broader socio-cultural forces, including media representations, commercial marketing strategies, and the gendered structuring of consumer environments, are also likely to influence parental behaviors. While our findings underscore the distinct contribution of PAGC to the variability observed in gendered parenting, we do not claim to capture the full range of determinants underlying these complex socialization processes.

To evaluate the cross-cultural robustness of our hypotheses, PAGC was examined as a predictor of gendered parenting in two distinct contexts – Israel and the United States. Some variations were observed: for example, Israeli fathers showed more gendered parenting than mothers whereas in the United States there was no gender difference in gift selection. Moreover, PAGC exerted a slightly stronger effect on parental discomfort in the pajama scenario among American parents. Nonetheless, PAGC reliably predicted gendered parenting measures across both samples. This pattern corroborates the idea that parental aversion to the possibility of having a gay or lesbian child exists in both cultures, and although the behavioral expression of such concerns may vary according to contextual norms and expectations, it constitutes a significant factor contributing to gendered parental practices.

To further clarify the theoretical contribution of PAGC, it is important to distinguish this construct from other related concepts in the theoretical field. First, it differs from the concept of heteronormativity: although both share a mutual basic opposition towards same-sex orientation, heteronormativity lies within a cognitive framework of gender/sex views and beliefs, while PAGC represents more of an emotional stance, comprised of negative feelings like fear, concern, sadness, resentment, and even anger in response to the possibility that one’s child may develop a same-sex orientation. It is very likely that such emotional stances are one of the emotional consequences of the heteronormative view.

Indeed, despite far-reaching changes in recent decades and the growing openness towards different gender expressions and sexual orientations, especially in Western societies, the most prevalent and socially accepted model remains heteronormative [104]. It has also been argued that the entire educational system, and schools in particular, enforce and reproduce heterosexist, patriarchal ideology by basing everyday decisions regarding curriculum and social events on the assumption that human beings are inherently heterosexual [105]. Moreover, even in the face of social changes in masculinity norms, perceived men’s feminization was still found to invoke discomfort and negative attitudes toward same-sex orientation among heterosexual men [106]. Thus, parents who adopt conservative concepts of gender roles may also perceive heterosexual orientation as part of the accepted model of “femininity” or “masculinity” and therefore may resent their child behaving in a gender non-conforming way, as well as the possibility that he/she may develop a same-sex orientation.

A second important distinction should be made between parental aversion to the possibility of having a gay or lesbian child and homophobia. PAGC, unlike homophobia, is not rooted in prejudice against LGBT individuals [107]; nonetheless, both PAGC and homophobia contain fear based on a sense of apprehension toward something presumed to be harmful or dangerous [108]. Therefore, both constructs may be considered as part of the “homophobic system” – a combination of heterosexist beliefs and practices (including heteronormativity, sexism and male dominance) that maintain consistency between sex, gender and sexual orientation, while stigmatizing same-sex orientation as deviant [109]. Kimmel [110] has argued that “homophobia is a central organizing principle” of the American cultural definition of manhood, being dominated by the fear of “being seen as a sissy.” Indeed, previous research has found that men are reluctant to engage in activities stereotypically considered “feminine” because they fear they will be mistakenly “suspected” of being gay [111, 112]. Our findings indicate that to the same extent that adults, and heterosexual men in particular, possess this fear in relation to themselves, parents also hold it in relation to their children.

Parents’ responses in our studies also showed that they feel responsible for the gender socialization of their children and for teaching them about acceptable social norms. However, the assumption that parents can shape their children’s sexual orientation is questionable at best. While the exact causes of sexual orientation remain largely unknown, there is growing consensus that it is not something that can be externally shaped or changed through upbringing or intervention [113, 114]). Research has consistently shown that attempts to influence or alter sexual orientation, such as through gender policing or conversion efforts, are ineffective and can be harmful to children’s mental health and well-being [115].

Thus, parents who convey to their child that same-sex orientation (or non-conforming gender behavior, for that matter) is a negative, undesirable, or even obscene thing, may lead their non-conforming children to feel unloved, unwanted, and unaccepted by the people who are supposed to love them unconditionally. Such children and teenagers may feel that they must hide or be ashamed of who they really are, feel guilt and inauthenticity, experience distress, and be at risk of various psychological disorders, such as major depression, substance abuse, sexual risk behavior, and suicidal attempts (e.g., [116, 117]). On the other hand, parental support and acceptance of adolescence’s sexual minority (LGB) was found to be a resilience factor, protecting these children from mood disorders and drug use (e.g., [118]). Since our findings show that parental aversion to the possibility of having a gay or lesbian child is a clear predictor of gendered parenting practices, psychoeducational interventions can focus on increasing parents’ acceptance of diverse sexual orientations, and on addressing the potential harm of trying to direct children toward a specific path based on heteronormative expectations. Such interventions may, in turn, reduce gendered parenting practices and the parental pressure exerted on children to conform to gender-compatible norms.

This research has several limitations. First, its correlational design prevents drawing causal conclusions regarding the processes underlying gendered parenting. Future research could establish causality by manipulating views of social hierarchy and essentialism, as demonstrated in prior experimental studies (e.g., [59,119]). Second, the studies did not account for children’s preferences, which are likely to influence parenting choices. However, our focus on parents’ deep ideological orientations and emotional stands suggests that these motivations significantly drive gendered parenting beyond the impact of children’s desires. Third, additional parental variables, such as education level [14] and family structure [120] were not addressed and should be explored in future research to provide a more comprehensive understanding of gendered parenting.

Lastly, a notable limitation of the present research is its exclusive focus on cisgender, heterosexual parents. While this decision enabled a more focused exploration of gendered parenting within the most studied demographic group, it limits the applicability of the findings to more diverse family structures. Prior studies indicate that LGBTQ+ parents may differ in their approaches to gender socialization – for example, by fostering more egalitarian environments or practicing gender-open parenting – highlighting the need for further research in this area [95–97]. Future studies should therefore examine gendered parenting across a broader range of sexual orientations and gender identities [121,122].

In light of these limitations, another important avenue for future research concerns the broader expressions of gendered parenting and the factors that contribute to them. The present study focused on the selection of gender-typed toys as one possible manifestation and the chosen operationalization of gendered parenting. However, parental aversion to the possibility of having a gay or lesbian child may influence a broader range of socialization practices, such as parental encouragement of same-sex peer relationships, reinforcement of gender-stereotypical activities, or shaping educational and occupational aspirations. Thus, PAGC may contribute to various facets of gendered socialization beyond the specific behaviors examined in the current study. Future research could usefully explore additional expressions of gendered parenting and further elucidate the broader socialization patterns associated with parental concerns regarding their child’s (future) sexual orientation.

To further explore gendered parenting, an additional factor that may provide more context for understanding our findings is gender norms. Borinca et al. [112] showed that perceived social changes in men’s gender norms (i.e., the notion that “feminine” behavior is becoming more common and socially accepted among men) calmed the fear of being classified as gay and, in turn, reduced the level of discomfort felt by the men. Stated differently, when men were asked to imagine themselves, for example, dancing in a ballet class or shopping for clothes with friends and were led to believe that the norms of masculinity are changing, they were less worried about being perceived by others as gays and thus felt less embarrassment and discomfort [112]. The manipulation of gender norms in this study (there, Study 3) referred to masculinity as either something that remains constant and stable or something that is culturally defined and changes over time.

This suggests that perceived changes in gender norms can be a source of change in the studied processes, such that if parents come to view masculinity as a more malleable construct, they might be less aversive to the possibility of their children developing a same-sex orientation. Parents can also be taught about less binary gender perceptions, showing that individuals, both women and men, are composed of a mosaic of traits and characteristics. This “gender mosaic” notion is supported by findings in brain research [123] and personality research, showing that most individuals have a mix of both (stereotypically considered) “feminine” and “masculine” characteristics [124,125]. In conclusion, psychoeducational interventions among parents, aimed at affecting their gender views, along with the emotional aspects of fear and resentment towards children’s gender deviancy, might be an effective path in reducing gendered parenting – allowing for children’s exposure to a variety of fields and experiences, hopefully leading to greater self-realization and prosperity for every child.

## Supporting information

S1 File Measures’ details. (DOCX)
