# Supplementary material for: Parents’ aversion to the possibility of having a gay or lesbian child predicts gendered parenting
Source: PLoS One. 2025 Dec 4;20(12):e0338209. doi: 10.1371/journal.pone.0338209 (PMC12677495; doi:10.1371/journal.pone.0338209)
Supplement: S1 File — (DOCX) [file pone.0338209.s001.docx]

**Parents' aversion to the possibility of having a gay or lesbian child predicts gendered parenting**

**Supplementary materials**

**Method**

**Measures**

Same measures and procedure were used Study 1 and Study 2, except these few differences: (1) In Study 1 all measures were in Hebrew, in Study 2 all were translated to English, and we used an adapted version of the GTC measure, to suit American parents (see below). (2) In Study 2, we used an additional measure of gendered parenting, by which parents were asked to choose their most preferable activities for their child. (3) In Study 2 we did not measure SDO. Thus, unless otherwise stated, the following measures were used in both studies.

**Gendered Parenting Measures (DV)**

***The Gendered Toy Choice (Hebrew Version; Study 1)***

Table 1 presents the type of toys presented to the parents, and the gender-typicality score of each toy (based on the pretest ratings), that was used to code the Index score. In the study, the toys were presented to parents by pictures, in a mixed-order matrix.

*Table 1*

Toys presented to the Israeli parents in Study 1, and the gender-typicality score of each toy used for coding the Index (based on pretest ratings*; N=82*).

| Gender-typicality score  (ranging from 1- extremely feminine;  to 9 - extremely masculine) | Toys |
| --- | --- |
| 1.17 | Make up set |
| 1.22 | Barbi doll |
| 1.26 | Big pink doll |
| 1.29 | Hair decoration kit |
| 3.06 | Mandala creation kit |
| 3.77 | Kitchen oven |
| 3.82 | Stove |
| 3.89 | Groceries trolly |
| 4.38 | Play-Doh |
| 4.87 | Building straws |
| 5.23 | Mr. potato |
| 5.26 | Gobblet- Gobblers game |
| 6.88 | Offbits (robot creation kit) |
| 6.93 | Cars' magnet creator |
| 7.32 | Table basketball |
| 7.62 | Airport kit |
| 8.06 | Remote control car |
| 8.11 | Transport Truck (with small cars) |
| 8.49 | Tobot Robot |
| 8.51 | Avengers action figure |
|  |  |

***The Gendered Toy Choice (English Version; Study 2)***

Table 2 presents the type of toys presented to the American parents, and the gender-typicality score of each toy (based on the pretest ratings; *N=80*; and additional sample of parents which rated few mor toys, *N=68*), that was used to code the Index score. In the study, the toys were presented to parents by pictures, in a mixed-order matrix.

*Table 2*

Toys presented to the parents in Study 2, and the gender-typicality score of each toy used for coding the Index (based on pretest ratings).

| Gender-typicality score  (ranging from 1- extremely feminine;  to 9 - extremely masculine) | Toys |
| --- | --- |
| 1.23 | Hair decoration kit |
| 1.24 | Make up set |
| 1.29 | Barbi doll |
| 1.40 | Big pink doll |
| 2.79 | Stove |
| 3.41 | Kitchen oven |
| 3.94 | Groceries trolly |
| 4.46 | Mandala creation kit |
| 4.95 | Building straws |
| 5.09 | Play-Doh |
| 5.13 | Gobblet- Gobblers game |
| 5.41 | Mr. potato |
| 6.39 | Cars' magnet creator |
| 6.47 | Table basketball |
| 6.63 | Offbits (robot creation kit) |
| 6.71 | Airport kit |
| 7.71 | Remote control car |
| 7.89 | Transport Truck (with small cars) |
| 7.91 | Tobot Robot |

***Pajama Scenario - Explicit dis/encouragement of child's counter-stereotyping behavior***

A single imaginary scenario item (gendered parenting behavioral intentions in face value). Parents chose their response to their child request to get as a present and try on a pajama in a counter-stereotypic design (different version is presented to parents to boys vs. parents to girls).


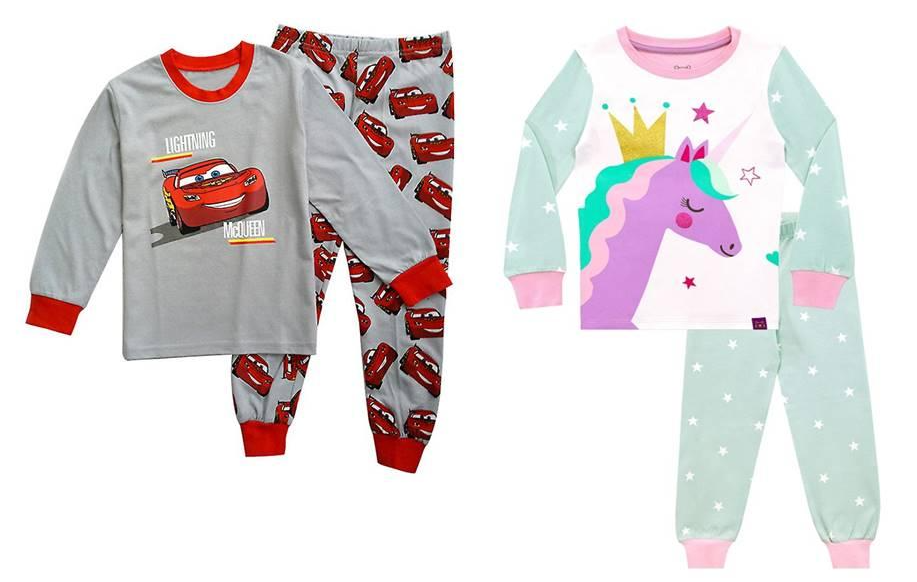
The parents in your child's kindergarten have decided to buy a pajama suit for all the kids in the kindergarten. The pajama will be handed to each child on the day of their birthday celebration, in which the child will open the gift and will try on the new pajama. Parents were given a choice between these 2 pajama suits:

**Imagine your son (daughter) saw these options and asked you to choose**

**the unicorn (cars) pajama for his (her) birthday**. How do you think you will respond?

1. Excellent! I will praise him (her) for his (her) choice
2. That's perfectly fine with me, I'll let him (her) choose and wear it in kindergarten
3. I am willing to accept it
4. I don't mind
5. I'm not sure it's a good idea, maybe I'll be able to convince him (her) to choose the other option
6. It's not a good idea, I wouldn't want him (her) to wear it in the kindergarten
7. No way! I will explain to him (her) that I think this is inappropriate

If you like to explain your response - we would appreciate it if you could explain here: _____________________________________________________________^[[1]](#footnote-1)^

***The Gendered Activity Choice (Study 2)***

Table 3 presents the activities presented to the American parents, and the gender-typicality score of each activity (based on the pretest ratings), that was used to code the Index score. In the study, the activities were presented to parents in a mixed-order matrix, in which they had to mark 3 boxes of their preferred activities for their child.

*Table 3*

Activities presented to the American parents in Study 2, and the gender-typicality score of each activity used for coding the Index (based on pretest ratings).

| Gender-typicality score  (ranging from 1- extremely feminine;  to 9 - extremely masculine) | Activities |
| --- | --- |
| 1.96 | Ballet dancing class |
| 2.13 | Knitting class |
| 3.12 | Yoga class |
| 3.33 | Aerobics class |
| 3.90 | Cooking class |
| 4.01 | Ceramics class |
| 4.53 | Origami (paper folding) class |
| 4.58 | Paper mash class |
| 4.79 | Drawing class |
| 5.10 | Capoeira class |
| 5.24 | Math fun class |
| 5.53 | Ping-pong (table-tennis) class |
| 5.56 | Computers class |
| 5.66 | Lego class |
| 5.70 | Soccer class |
| 5.76 | Chess class |
| 6.40 | Basketball class |
| 6.40 | Judo class |
| 6.55 | Dungeons & Dragons class |
| 7.06 | Ninja class |
| 8.26 | Football class |

**Measures of the Predictors**

***Endorsement of Social Hierarchy*** ***(SDO; Pratto et al., 1994) (Study 1)***
 Support for group-based hierarchy was assessed with an adapted, 8 items' version of the Social Dominance Orientation Scale.

1. Group equality should not be our primary goal.
2. It is stupid to try to make groups equal.
3. We should do what we can to equalize conditions for different groups (R).
4. We should work to give all groups an equal chance to succeed (R).
5. Some groups of people are simply inferior to other groups.
6. An ideal society requires some groups to be on top and others to be on the bottom.
7. Groups at the bottom are just as deserving as groups at the top (R).
8. No one group should dominate in society (R).

***Gender Essentialism (Skewes et al., 2018)***

In both studies we used an adapted, shortened 7 items' version of this measure.

Please indicate to what extant you agree with the following ideas (1- strongly disagree; 7- strongly agree):

1. Differences between women and men’s personalities are in their DNA.
2. Differences between men and women in behavior and personality are largely determined by genetic predisposition.
3. Differences between boys and girls are fixed at birth.
4. Male and female brains probably work in very different ways.
5. Differences between men and women are primarily determined by biology.
6. Upbringing by parents and the social environment have far greater significance for the development of sex differences than inborn differences in female and male brains (R)
7. People tend to be either masculine or feminine: there’s not much middle ground.
8. It's important that you pay attention to this study. Please tick 'Strongly disagree' (*Attention check item)

***Child Rearing Gender Ideology (adapted from Burge, 1981; Freeman***[***2007***](about:blank#ref-CR23)***; Endendijk, et al., 2013)***

Now please relate to your attitudes regarding raising children, and indicate to what extant you agree with the following ideas (1- strongly disagree; 7- strongly agree):

1. Quiet girls will have a happier life than assertive girls.
2. Boys who exhibit sissy behaviors will never be well adjusted.
3. Girls, more than boys, should care about well-groomed appearance.
4. I feel upset when I see boys put on a dress when they play dress-up.
5. Boys, more than girls, need competitive skills.
6. It makes me uncomfortable when girls play in little league soccer.
7. Boys should be encouraged to engage in therapeutic professions
   (a nurse in a hospital, a kindergarten teacher) (R)
8. Girls who are tomboys will never be well adjusted.

|  |
| --- |

***PAGC - Parental Aversion to the possibility of having a Gay or lesbian Child***

People have a variety of feelings and thoughts about sexual orientation. If your child had homosexual tendencies:

To what extent would you be worried or not worried?
(1- Not worried at all; 7- Very much worried)

To what extent would it be disturbing or not disturbing for you?
(1- Not disturbing at all; 7- Very much disturbing)

To what extent would it be upsetting or not upsetting to you?
(1- Not upsetting at all; 7- Very much disturbing)

To what extent would it make you happy or not happy?
(1- Not happy at all; 7- Very much happy)

To what extent would that be resentful or not resentful to you?
(1- Not resentful at all; 7- Very much resentful)

1. This item was used for the qualitative analyses. [↑](#footnote-ref-1)
